# Lectures on Experimental Physiology, Delivered at the College De France, Paris, during the Year 1855

**Published:** 1856-05

**Authors:** Claude Bernard


### SELECTIONS.

*Lectures on Experimental Physiology, delivered at the College de France, Paris, during the year* 1855. By Professor Claude Bernard. From Notes taken at the Lectures by Anthony Peniston, M.D., of New Orleans.

*Gentlemen*—The mode of instruction adopted in the College de France differs materially from that of a Chair of Physiology in a Medical Faculty. In the latter, the teacher must promulgate his opinions dogmatically, considering the science as settled and complete. Here, on the contrary, we must consider every question as being open to investigation, and the science of physiology itself as if it were imperfect and unsettled. They must search among the records of the past, and must build up their fabric from the labors of others: we must look on the future destinies of our science. Let ours be the hope of extending its limits. Two methods of contributing to the progress of Physiology now present themselves:—

1st. To make new researches.

2d. To criticise old doctrines by submitting them to the test of experiments.

The celebrated Le Maistre said, that more discoveries were due to chance than to all the researches of the learned; he even said, that in order to make new discoveries it was necessary to be ignorant.

On the other hand, Bacon and his school have contended, that there is a method with certain rules by which the limits of science can be extended and new discoveries made.

That those principles are useful to guide us in our researches there can be no doubt; notwithstanding, experience has not proved that those who knew them best were the most fortunate in their application.

During the course of these lectures we shall investigate the mode of action of divers substances, which exercise a notable influence on the phenomena of life.

Two conditions are requisite to maintain life. 1st. The organism, that is to say, the integrity of all the organs, of the tissues, and their functions. 2d. The medium in which we live, comprising oxygen, heat, and the food requisite for our organism. But, at the same time, that we must study those chemical changes necessary to maintain life, so also must we investigate the nature of many substances, which, though not necessary to life, yet may be said to exercise a marked influence on our organization, suspending or destroying its functions.

Thus the numerous class of poisons and medicaments are perturbing agents, while oxygen, on the contrary, may be styled a conservative agent. But the study of medicines and poisons constitute the domain of therapeutics and toxicology.

Here we shall select a few of the most active substances, such as strychnine, nicotine, morphine, &c., in order to study their effects under a purely physiological point of view. We shall make use of them, as of a re-agent, which, by a specific mode of action, localized in some one tissue, will enable us to acquire precise notions on the nature of those tissues thus acted upon.

Physiology has no other aim or object than to analyse the phenomena of life.

In order to distinguish the part which appertains to each organ, amid the complicated phenomena of life, it has occurred to physiologists to destroy one organ, or tissue, and then to observe how the organism is affected by its absence.

Thus, by cutting a nerve, we destroy the functions of that nerve, and from it results either loss of motion or of sensibility, according as it is a nerve of motion or sensation.

But this process is exceedingly rude, and then when it becomes necessary to penetrate very deeply into adjoining structures in order to destroy some special organ, the morbid process may become so complicated as to involve the result in considerable obscurity.

When we examine the poisonous effects of certain substances, we ascertain that they act by destroying some one system of the organism, or some one tissue of the body. This point once settled thus becomes an excellent means of ascertaining the properties and functions of the different tissues. Thus, a substance of which the composition is not well known, but which possesses most active properties—the celebrated Woorara poison—exercises a particular action on the nervous system, it produces death without any apparent suffering, the animals manifesting no pain.

If we take two frogs, and sacrifice one by cutting off its head, we shall observe that, at a low temperature, the nervous system retains its properties during several days, and may react on the muscles under the influence of any exciting cause, such as galvanism or electricity.

On the other hand, if we poison the other frog with the Woorara poison, the excitability of the nervous system ceases at once: this poison annihilates the nervous system, and death is caused by the total abstraction of the nervous influence.

Now, it is utterly impossible to remove by dissection the whole of the nervous system; at the same time we should lacerate and destroy all the surrounding tissues. But here we have a substance following the nerve in its minute ramifications to its ultimate fibrilla, at the same time that it respects completely the surrounding organs.

But let us return to our experiment of the two frogs. On the one poisoned with Woorara, galvanism produces no effect on the nervous system, yet when applied locally to the muscles it produces contraction. On the contrary, if on the decapitated animal we isolate a nerve, and touch that nerve with a galvanic current, we observe convulsive movements in the whole limb. This experiment proves that Woorara destroys at the same time the motor and sensitive system of nerves, but that it does not affect muscular irritability. Thus the question mooted by Haller, more than a century ago, that muscular irritability is independent of the nervous influence, is now finally settled. Marshall Hall still maintains that nervous excitability and muscular irritability depend one on the other.

But let us go one step further. The experiment with the Woorara poison proves, moreover, that the irritability of the muscular system is increased by the effect of the poison, and persists nearly twice as long as it does on the decapitated animal. But it may be objected that no two frogs can be in the same identical condition, and that if the experiment had been reversed, the result would also have been different. The following experiment must remove all objection of that nature. Let us take a frog, and while it is in perfect health we put a ligature around the femoral artery on one side, and then poison the animal with Woorara. Now, it follows that the blood cannot carry the poison to that leg, and what is the result? That muscular irritability disappeared at the end of five days in the leg where the poison does not circulate, while it persisted ten days in the other leg. We may then safely conclude that the Woorara poison destroys nervous excitability at the same time that it increases muscular irritability.

There is yet another consequence to be deduced from our experiment. Let us examine the heart of the frog poisoned with Woorara; you will observe that it still beats perfectly. This proves that the heart is independent of all nervous action; and is in that respect quite an exception to the general rule. This is the reason that though separated from the brain and spinal marrow the heart still contracts.

You perceive from this experiment how we can make use of poisons to analyze the phenomena of life, they become, in the hands of the experimental physiologist, a re-agent, exercising a determinate action on certain parts of the organism, just as the chemist uses his tests in the laboratory to analyze and separate different substances. If it be asked what science we would promote, what good can result from our researches; we should answer, that this art is especially destined to throw light on medical science. It has been said that medicine and physiology are independent sciences, that the latter is more curious and entertaining than useful to the physician; for, say they, to what physiological state does fever correspond? To that we should answer, that if physiology was more cultivated we should then know what particular morbid state or derangement of which set of organs the fever was owing to.

Thus it is we know at present that diabetes has its physiological correlative. A few years ago, if it had been asked, is diabetes the result of some functional disorder producing sugar as a substance entirely foreign to the system, or is it produced by some mal-assimilation of the digestive organs, what would have been the answer? At this day, we know that the presence of sugar in the urine is owing to the exaggeration of a strictly physiological function, for in the normal state sugar is secreted by the liver. We know, moreover, that we can render animals diabetic by introducing a sharp pointed instrument into a certain portion of the medulla oblongta.

Fever also can be produced by some functional disorder. Thus, if we cut certain *filaments* of the great sympathetic nerve, fever supervenes in that part. (Here Mr. Bernard shows a rabbit on which he has made a section on one side of the sympathetic nerve distributed to the ear, and the result is plainly visible in the increased heat and redness of that ear compared with the other.) It has also been ascertained that if a section be made of a certain part of the great sympathetic distributed to the thorax or abdomen, we can produce pleurisy in the first, and peritonitis in the second. Undoubtedly there are many morbid states which we cannot explain by physiological laws as yet known; but it cannot be denied that the progress of physiology is destined to throw great light on those pathological conditions which are now the most obscure.

---

### LECTURE II.

In order to show the relative bearings of physiology and pathology, it may not be uninteresting to give a short sketch of the differet theories and opinions on the nature of diabetes. This disease has been known from the most remote antiquity; but it was characterized in a very vague manner, and its symptoms very imperfectly described. Thus, a person was said to be diabetic when he voided unusual quantities of urine, accompanied with other general symptoms, such as emaciation and loss of strength. On account of the latter symptoms, it has been likened to phthisis, one being of the lungs, the other of the kidneys, but the presence of sugar in the urine was long unknown. When this peculiarity was ascertained, a distinction was made between simple diabetes, or polyuria, and diabetes mellitus. In the present state of science this distinction is not admitted, diabetes being always characterized by the presence of sugar in the urine. The celebrated Willis was the first who said that the urine of diabetics was sweet and had the taste of honey. Rollo, who came afterwards, thought that the presence of sugar was due to a vitiation of the gastric juice, which changed the food into sugar instead of transforming it into chyle. Nicolas and Gueudeville, who wrote on the subject towards the year 1803, placed the seat of the disease in the intestines. They thought that chyle, being a nitrogenous fluid, produced by digestion, the diabetic sugar was nothing more than chyle deprived of nitrogen. In 1813, Mr. Chevreuil showed that diabetic sugar was similar to that produced from starch, which is transformed into sugar by the process of digestion.

Mr. Bouchardat thinks that sugar is formed in the stomach, and that it is absorbed directly. Mr. Mialhe has propounded another theory. Having ascertained that when diabetic sugar, or sugar produced from starch, is heated in presence of an alkali it is completely destroyed, Mr. Mialhe thought that sugar was a normal product of digestion, and that if the blood was not sufficiently alkaline to destroy the sugar, the person became diabetic; therefore, said he, the cause of the disease is in the blood, from which he concluded that the treatment should be by means of alkaline remedies.

But setting aside all these conflicting theories, formed, most of them, *a priora*, when we undertake to account for the presence of sugar in the urine, we find that it can only have its origin in two ways. Either it is introduced as food, or is fabricated abnormally in the system. We shall endeavor to show that in the normal state we secrete sugar internally, but that physiologically this sugar disappears before it reaches the kidneys. Diabetes, then, is nothing more than a deviation of a physiological state. Before considering the origin and production of sugar in the system, we must premise that chemists recognize three different kinds of sugar, viz.:—

Cane sugar.
Grape sugar.
Starch sugar, or glucose.

Cane sugar differs from the others in this, that the alkalies have no action on it, but the acids transform it into grape sugar, or glucose.

The alkalies have a particular action on grape sugar and glucose—thus, if we take a solution of potassa, and add to it some diabetic sugar or glucose, and then expose it to heat, the liquid takes a brownish hue, the sugar disappears and is transformed into various acids. On the contrary, ordinary cane sugar, being heated with potassa, does not alter its color. Pure cane sugar, boiled with an acid, is transformed into grape sugar. The following test is considered one of the best to detect the presence of sugar in the urine: Take a solution of tartrate of potassa and copper, with an excess of potassa, put a few drops into a test tube containing diabetic sugar, and expose the whole to the flame of a spirit lamp; the liquid, which is previously of a beautiful blue, speedily changes color, giving rise to an orange yellow precipitate.

Cane sugar, exposed to the same test, gives no result, as it exercises no chemical action on the tartrate of copper. Being exceedingly delicate, this test can be used to ascertain the proportion of grape sugar contained in a given solution. When we use the test for grape sugar, in the way above mentioned, the reaction which takes place is called a reduction of the liquid. The chemical change which takes place is as follows: When either grape sugar or glucose is heated they are converted into various acids. Now, this liquid tartrate of potassa contains a salt of duetoxide of copper, and when the sugar is decomposed, it takes away one equivalent of oxygen from the copper, which thus becomes a salt of protoxide of copper, and that being insoluble is precipitated. This liquid, which is known under the name of Barriswill's test, only gives this result when in presence of grape sugar or glucose. Potassa has also been used as a test, but it may lead us into error, as a solution of tannin heated with potassa gives the same brown color as a solution of glucose.

When the liquids, which we wish to test for sugar, are colored—as blood, for instance—we must filter them through bone black; this takes away the coloring matter and the other albuminous and organic substances, but in no way affects the sugar; we thus obtain a pure and transparent liquid, which can be readily tested by the method above mentioned. Another method, which allows us to ascertain the presence of sugar, is to produce fermentation by means of yeast, the sugar being decomposed into carbonic acid and alcohol, the latter burning with a peculiar bluish flame.

Polarized light has also been used to distinguish certain characters appertaining to grape and cane sugar.

Having thus explained the different kinds of sugar, and the tests by which it can be recognized, we are now prepared to investigate the origin and production of sugar in the system.

We have ascertained, beyond a doubt, and our experiments will prove, that sugar is formed from the blood, but that its production is totally independent of the species of food taken into the stomach. No sugar is found in the blood before entering the liver, but its presence in the blood which comes out of the liver is easily ascertained. The liver itself contains sugar in considerable quantity, and is the only organ possessing that property. It was long thought that the presence of sugar in the urine of a diabetic person, was owing to imperfect digestion of some particular kind of food; hence, physicians directed their exclusive attention to the diet of those patients, until it was almost universally admitted that all those substances which contained starch in any form increased the disease. Now, however, we must place the disease in some functional disorder of the liver, the nature of which we shall endeavor to elucidate in the progress of these lectures.

The existence of sugar in the liver being settled beyond all doubt, it was and is still objected by some physiologists, that this sugar did not take its origin in the liver, but having been introduced through the medium of absorption, was only localized and concentrated in the liver. That a dog, for instance, having eaten bread or some other substance containing starch, which is capable of being transformed into glucose, would have this sugar carried to the liver by the general circulation, and there it would be liable to remain a considerable time. A great deal has been said of this localization of different substances in the liver; thus it has been reported that mercury, copper, arsenic, &c., had been found in the liver of persons who had taken those substances during life. Therefore, some physiologists, reasoning by analogy, said it would not be strange if the sugar had reached the liver in the same way. But whatever may be the fact as to those other substances, the best proof that sugar is not introduced in that way, is, that we find it in the liver of the fœtus before any food has been taken into the stomach. Here are several livers taken from calves at the fourth or fifth month of gestation; you see that our test plainly indicates the presence of sugar: moreover, when submitted to the process of fermentation, alcohol will be produced. Certainly it cannot be said that this sugar was introduced by means of the food, for the stomach had never received any.

It might be said that the mother takes in food, and that this sugar may be introduced into fœtal circulation by vascular communication through the placenta. But it is well known that no such communication exists between the mother and the fœtus; moreover, the blood globules of the fœtus are larger than those of the mother, and could not circulate in the capillary vessels of the latter.

Another fact comes in to settle the question. The function of the liver which produces sugar, does not begin until the fourth or fifth month of uterine life; before that period no sugar is found in the liver, though the same relation exists between the mother and the fœtus, as far as nutrition is concerned, before that period as well as afterwards.

There is yet another proof that sugar is not simply localized in the liver; for those substances, such as arsenic, mercury, &c., which have been said to be found in the liver, when once they penetrate into the liver, remain there indefinitely. Sugar, on the contrary, disappears from the liver as soon as the function of the liver ceases. Thus, when animals die of disease, their liver contains no sugar. This is the reason why no sugar is found in the liver of the subjects commonly brought to our amphitheatres.

Although there be no relation of cause and effect between the nature of the food and the formation of sugar in the liver, yet innervation exercises a direct influence on this function. When a section is made of the pneumogastric nerves, the formation of sugar in the liver ceases at once. Here is a rabbit on which were cut those two nerves yesterday evening: you see he has survived the operation; we shall now sacrifice him, in order to prove that all traces of sugar have disappeared from the liver.

We must therefore admit that sugar is formed in the liver through a special function appertaining to that organ, and subject to certain physiological variations, according to the state of the system.

2d. That this function commences at a certain period of uterine life.

3d. That, during disease, the formation of sugar either diminishes or ceases completely. This is an excellent method of ascertaining whether an animal died in a state of health or in consequence of disease, for, in the former case, sugar would be found in the liver, and in the latter not.

Here is the result of an analysis, giving the proportion of sugar found in the livers of different animals:—

Human fœtus, at 6½ months,.............0.77 per cent.

Fœtus calf, from 7 to 8 months,.........0.80 "

Fœtus of a cat, at term,...................1.27 "

On the other hand, the following analysis shows that the different varieties of food exercise very little influence on the production of sugar in the liver:—

A dog fed exclusively on meat,.....1.90 grammes per cent.
Another fed the same,...............1.40 " "
A dog fed on meat and bread,.....1.70 " "
Another fed the same,...............1.30 " "
A dog fed exclusively on starch,...1.88 " "
Another fed in the same way,......1.50 " "

The liver, you know, is a large glandular structure, situated in the abdomen, and placed as a sort of barrier between the digestive system on the one hand, and the general circulation on the other. The liver receives, by the vena porta, the blood which comes from the digestive organs; this blood traverses the liver, undergoing divers modifications, and then comes out by the hepatic veins, which empty themselves into the inferior vena cava. Besides these two sets of vessels, we have the lymphatics and the hepatic artery. The latter exercises no influence on the function of the liver, but only serves for the nutrition of the substance of the liver, and does not afford materials for the different products of that organ. Sugar is formed from materials furnished by the blood of the vena porta; this production of sugar is a secretion analagous to others in the animal economy.

Thus we see that several functions appertain to the liver, for we know already that it produces bile. The liver is an organ which exists in all animals. For a long time it was thought that the liver only served for the production of bile; and, as it was said by physiologists, that bile was useless in the animal economy, the argument was that the liver itself was unnecessary.

But, in the first place, it is now well ascertained that bile has most important functions in the process of digestion, and, moreover, the liver fulfills other purposes connected with the respiratory functions through the production of sugar.

It is said, that an organ which secretes, must be provided with an excretory duct. Now that is true so far as regards the secretion of bile, but there is no special duct for the elimination of sugar. It mixes with the blood coming from the liver, and is thence carried to the lung.

How does this secretion take place? All secreting organs are formed of cells, and this is the case with the liver. These cells are disposed in masses, which, by compression, assume a spheroidal or polyhedral form, constituting the lobules of the liver. At the centre of each lobule the commencing radicles of the hepatic vein take their origin; the branches of the vena porta, on the contrary, ramify on the surface of the lobules. The biliary ducts commence within the lobules by numerous ramifications, which form a closed network or plexus, occupying principally the outer portion of each lobule. The blood which enters the liver undergoes very important modifications in traversing the cells situated between the ultimate ramifications of the vena porta and the origin of the hepatic veins. There it is that the blood is divided into those component elements which produce sugar on the one hand and bile on the other. But these two secretions are entirely distinct, for there are two sets of cells, one forming the bile and the other the sugar; the bile being composed from the nitrogenous elements of the blood; the sugar, on the contrary, from the albuminous or non-nitrogenous elements.

The production of those two substances is not concomitant—that is to say, when the formation of bile is most active, that of sugar is at its lowest point, and *vice versa.*

The exact relation of the biliary ducts to the cells of the liver has never been well ascertained. Though coming from an organ containing sugar, the bile itself contains none, which proves that the two excretions are independent.

In the mollusca, snails, and others of that class, it can be seen that the bile, or rather the liquid coming from the liver, does not always present the same character. When digestion is going on, the duct which comes from the liver furnishes a very sweet liquid; then this liquid ceases after a certain time—it changes color from white to green, becomes exceedingly bitter, and no longer retains any traces of sugar. Thus the production of sugar takes place during digestion; that of bile, on the contrary, takes place after digestion. This is contrary to the old admitted opinion, that bile was produced during digestion. But, that this is not the case, can be easily demonstrated on a dog having a biliary fistula. Here is the operation which must be made to establish a biliary fistula on that animal. Make an incision in the right side, enabling you to pass a ligature around the ductus communis choledochus, which is thus prevented from emptying into the abdomen. You must then draw out the gall bladder, and attach it to the skin, where it becomes adherent; lastly, you make a small puncture, and then the gall bladder itself becomes an excretory duct, by which the bile runs out. By this means you can ascertain that, during digestion, bile runs in very small quantities; but that about seven hours after a meal, much larger quantities of bile are excreted. During digestion bile is poured into the intestinal tube, but it comes from the gall bladder, into which it has been accumulated beforehand, as a sort of reservoir. Sugar, on the contrary, is produced about three hours after an animal has eaten.

All animals are not alike provided with a gall bladder; the horse, for instance, has none, but the ductus communis choledochus is very large, and provided with a sphincter, which thus retains the bile.

In certain insects the liver does not exist as a distinct organ, but we find in its stead two series of glands, of which those situated above secrete bile, and those below secrete sugar, while both pour their secretions into the intestinal tube. We have thus far arrived at the following conclusions, which may be stated as general propositions.

1st. There are two functions in the liver, but they do not seem to be the result of the same anatomical element—one producing sugar, the other bile.

2d. These two secretions are neither permanent nor constant, but intermittent, there being oscillations in the secretion of sugar as well as bile.

3d. Sugar is not an excretion like urine, for in the physiological state it should not appear outside the system; it is only pathologically, as in diabetes, that sugar is excreted with urine. The following is the origin and progress of sugar in the system: After its production in the hepatic cells, sugar comes out of the liver by the hepatic veins; it then reaches the inferior vena cava, where it mixes with the blood coming from the lower extremities; from the inferior vena cava, the sugar passes into the right side of the heart, whence it is driven into the lung. There, the sugar is brought into contact with the atmospheric air, and is destroyed by undergoing a peculiar species of combustion, so that when the blood comes out of the lung it no longer contains any sugar. The quantity of sugar is at its summum in the interval between two meals, when digestion may be said to be most active; so that, though the secretion be constant, there are periods when it is more considerable than others.

The proportion of sugar in the blood of the hepatic vein is about one per cent., but in the right ventricle (and it has been still further diluted) the proportion is about $\frac{1}{3}$ per cent. At the beginning of digestion, the blood of the vena porta is supplied on the one part from the capillary terminations of the mesenteric arteries, and on the other from divers soluble matters and liquids absorbed in the intestinal tube. On this account the liver receives, at that moment, a much larger quantity of blood than during abstinence, the secretion of sugar is rendered more active, and then the proportion of sugar in the hepatic vein may be as much as 1½ or 2 per cent. The quantity of sugar being thus considerably increased, it may happen that all will not be destroyed in the lung, and then arterial blood will contain a small portion. This is generally found to be the case where an animal is sacrificed during digestion, for, whatever be the food, some sugar will be found in the whole circulation, but always in much greater proportion in the blood of the hepatic vein and vena cava. This ocillation, in the production of sugar, is very important, as it is found even in the pathological state, for it has been observed that some persons are diabetic at certain times and not at others.

The appearance of sugar in the urine may depend upon two causes; either its destruction in the lung is not sufficiently rapid, or its production is abnormally increased.

It being thus settled, that the sugar takes its origin in the liver, while, on the other hand, it is destroyed in the lungs, we perceive at once that a most intimate physiological relationship must exist between those two organs; and pathological anatomy has verified this law, for diabetic subjects have generally important lesions in the lung as well as in the liver.

In the physiological state, bile never contains any sugar. If you sacrifice an animal and examine the bile instantly, it will not contain any traces of sugar. But, on the other hand, if you were to examine bile taken from an animal which has been dead for some hours you may find evident traces of sugar. The reason is this: when life ceases, the liver becomes subject to purely mechanical laws, and as sugar has a strong endosmotic power, that which is contained in the hepatic cells may easily pass into the biliary ducts, whereas during life this is never the case.

In studying the functions of the liver, the following propositions present themselves for investigation:—

1st. What alterations may supervene pathologically in the cells of the liver?

2d. What changes take place in the composition of the blood while passing through the liver?

3d. What influence is exercised on the functions of the liver by the purely mechanical phenomena of the circulation?

4th. What is the influence of the nervous system on the functions of the liver?

As to the nature of the alterations which the hepatic cells may undergo, we know but little. The fatty liver is observable among certain animals fed in an artificial manner, producing a metamorphosis in the cells of the liver which are filled with large quantities of fat. The liver, however, still continues to secrete sugar, two analyses presenting, the one case 1, the other $1\frac{1}{2}$ per cent. of sugar, which is even beyond the ordinary proportion of sugar in the liver. Besides this, the liver is subject to a great variety of degenerations and morbid productions; but these alterations generally leave a portion of the organ in a healthy state, and this continues to supply the wants of the economy.

On the influence exercised by the composition of the blood on the production of sugar in the liver: —

As the composition of the blood in the vena porta varies according to the nature of the food, it is important to know what influence those changes may exercise on the production of sugar. This question has been raised especially in the treatment of diabetes, where it may be useful to know what kind of food produces the least sugar. Formerly physiologists thought that sugar in diabetes was produced exclusively by vegetable food, and consequently the deduction that those patients would be most benefitted by nitrogenous articles of diet and animal food, while, on the other hand, bread was supposed to furnish a large proportion of sugar. But as we have now ascertained that the production of sugar exists in the liver, what becomes of the sugar introduced into the system from outside?

Before answering this question, we must know what would be the consequence of prolonged abstinence on the production of sugar.

When an animal is entirely deprived of food, we have ascertained that sugar gradually disappears from the liver. Prior to this, however, sugar can only be produced from the materials furnished by the blood contained in the vena porta, for, in the absence of all food, the intestines can furnish no materials to the vena porta through the different absorbent vessels; therefore the blood, circulating in the vena porta, is furnished exclusively from the blood of the mesenteric arteries passing into the portal circulation through the intermedium of the capillaries. Thus we prove, that the production of sugar is independent of the nature of food. In an animal, exposed to complete abstinence, the liver continues to secrete sugar until the animal loses 4-10ths of its weight: at that period, there is a complete paralysis of the digestive organs, and if it were attempted to nourish the animal, its life could not be saved. Dogs, thus exposed to starvation, die from the twentieth to the twenty. second day; Guinea pigs, from the twelfth to the fifteenth day-

Touching this question of prolonged abstinence, it may not be uninteresting for us to learn what takes place in those hibernating animals which are known to pass whole months without eating. Valentin, who has experimented on the marmot, an animal inhabiting the higher regions of the Alps and Pyrenees, has ascertained that those hibernating animals are not in the condition of an ordinary animal deprived of food. Having kept one of these marmots, which slept 39 days without eating, he then sacrificed it, and found that the liver contained 2.87 per cent. of sugar. He found in the intestinal tube of those animals a peculiar whitish substance, which was continually secreted by the stomach, in a manner peculiar to those animals. There was then re-absorption of this matter in the alimentary tube, and nutrition was thus kept up at the expense of the animal itself, for it continually diminished in weight. In our next lecture we shall consider the influence of food on the production of sugar.—*N. O. Medical News and Hospital Gazette.*

[To be continued.]

